# Low-temperature-dependent property in an avalanche photodiode based on GaN/AlN periodically-stacked structure

**DOI:** 10.1038/srep35978

**Published:** 2016-10-24

**Authors:** Jiyuan Zheng, Lai Wang, Di Yang, Jiadong Yu, Xiao Meng, Zhibiao Hao, Changzheng Sun, Bing Xiong, Yi Luo, Yanjun Han, Jian Wang, Hongtao Li, Mo Li, Qian Li

**Affiliations:** 1 Tsinghua National Laboratory for Information Science and Technology, Department of Electronic Engineering, Tsinghua University, Beijing 100084, China; 2 Microsystem & Terahertz Research Center, China Academy of Engineering Physics, Chengdu 610200, China

## Abstract

In ultra-high sensitive APDs, a vibrate of temperature might bring a fatal decline of the multiplication performance. Conventional method to realize a temperature-stable APD focuses on the optimization of device structure, which has limited effects. While in this paper, a solution by reducing the carrier scattering rate based on an GaN/AlN periodically-stacked structure (PSS) APD is brought out to improve temperature stability essentially. Transport property is systematically investigated. Compared with conventional GaN homojunction (HJ) APDs, electron suffers much less phonon scatterings before it achieves ionization threshold energy and more electrons occupy high energy states in PSS APD. The temperature dependence of ionization coefficient and energy distribution is greatly reduced. As a result, temperature stability on gain is significantly improved when the ionization happens with high efficiency. The change of gain for GaN (10 nm)/AlN (10 nm) PSS APD from 300 K to 310 K is about 20% lower than that for HJ APD. Additionally, thicker period length is found favorable to ionization coefficient ratio but a bit harmful to temperature stability, while increasing the proportion of AlN at each period in a specific range is found favorable to both ionization coefficient ratio and temperature stability.

In an avalanche photodiode (APD), ionization process is fundamental for multiplication. High energy electron or hole triggers ionization and generates new carriers, then the signal is significantly enhanced. However, ionization process in conventional homojunction (HJ) APDs is greatly interfered by scattering, especially phonon scattering. Ionization coefficient is a key parameter to reflect the ionization efficiency, which is defined as the average ionization times triggered by a single carrier within the unit length along the transport direction. Keldysh in 1965 formed an equation of ionization coefficient as shown in Eq. 1^1^,


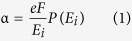


where 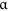 represents ionization coefficient, *e* represents electron charge, *F* represents electric field, 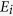 represents ionization threshold energy, and 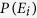 represents the possibility of electrons to gather enough energies to trigger ionization within the mean free path. 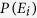 can be calculated by Eq. 2^1^


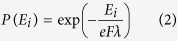


where 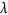 represents mean free path and 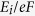 equals to the least accelerating length electrons need to gather enough energy without scatterings. Generally, the value of 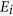 is fixed and 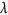 has a negative relationship with scattering rates, thus the scattering will restrict the ionization coefficient. In conventional HJ APDs based on commonly used materials such as Si, GaAs, InP, GaN, SiC, energy of electron or hole gathered from electric field is greatly thermalized due to phonon scattering^2^,^3^,^4^,^5^,^6^. The situation is shown in Fig. 1a, where GaN HJ APD is taken as an example. As a result, the ionization coefficient is not high enough. Furthermore, as phonon scattering is sensitive to temperature, the ionization coefficient is also strongly temperature-dependent. In order to achieve high gain, APD should work under extremely high bias, where both electron and hole could trigger ionization to form positive feedback ionization chain. In this case, the APD performance is highly sensitive to temperature. For example, in single photon detection, a vibrate of temperature might bring a fatal decline of the performance^7^. Even in some cases, heating could be used in blinding the APDs^8^. In many critical applications, APDs have to be mounted on a temperature maintained thermoelectric cooler to avoid temperature change^9^, which reduces the integration level of system.

Improving temperature stability has been a long lasting pursuit towards high performance APD. In the past, most efforts were put on improving the structure of APDs to achieve high temperature stability. For example, by using dead space effect, temperature dependent coefficient could be reduced through decreasing avalanche region width^10^. However, decreasing avalanche region width would bring in tunneling risk and increase noise^9^. In order to improve the temperature stability essentially, reducing scattering degree during electron transport is necessary.

Deformation scattering and optical polar phonon scattering are the two most important scattering types during electron transport. According to the Fermi-Golden rules, deformation scattering rate 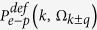 and optical polar phonon scattering rate 
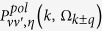
 from initial state at the point of *k* in band 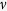 to final state at the point of *k* ± *q* (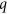 represents the phonon wave vector) in band 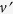 can be presented as Eqs 3–4^11^


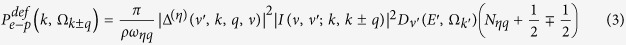



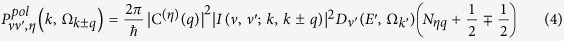


where 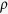, 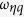 and 
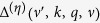
 represent crystal density, phonon angular frequency and deformation potential, respectively. 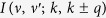 represents the overlap between wave functions of initial and final states. 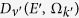 is a density of states (DOS) in the range of 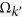 at 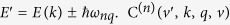 represents coupling coefficient of electrons and phonons. 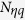 represents phonon occupation number and can be calculated by Eq. 5^11^


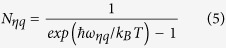


where *T* represents the temperature and 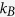 is Boltzmann constant. From the equations (3, 4, 5), it can be seen that deformation scatterings and polar phonon scatterings increases with temperature. Furthermore, if the DOS 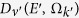 is low, the scattering rate will be reduced and become less temperature-dependent. If electron always transport in states with low density, the temperature stability would be improved, while the ionization coefficient stays high at the same time.

In GaN and AlN, there is a deep (~2 eV) separated 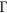 valley in first conduction band (CB) where the DOS is low^12^. However, the deepness of such 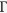 valley is lower than the bandgap of GaN, electron had to transfer to higher energy valleys to trigger ionizations, where the DOS drastically rises. Therefore, in conventional GaN based HJ APD, the ionization coefficient of electron is low (only about 1 × 10^4^/cm even under the high breakdown electric field of 3.2 MV/cm^5^). Here, we utilize an GaN/AlN periodically-stacked-structure (PSS) to solve the problem as shown in Fig. 1b. As there is a 2-eV CB offset between GaN and AlN^13^, with proper design of the periodical structure, before electron gets out of the 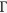 valley in GaN, it comes to the interface between GaN and AlN and gets back to 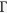 valleys of AlN until gathering enough energy to come back to GaN and trigger ionizations. Thus, electron could always transport in 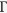 valleys with low DOS and we predict such an APD to be high temperature stable. Recently, we demonstrated GaN/AlN PSS APD to hold high linear gain^14^. In this paper, we focus on exploiting the high temperature stability potentiality for GaN/AlN PSS APD. It should be noticed that not all kinds of such periodically stacked structure could improve temperature stability. The deep Γ valley and large CB offset of GaN/AlN system provide an excellent material platform to realize high temperature stability for high sensitive PSS APD. Other material systems might not hold such performance.

## Results and Discussion

### General transport property

**Figure 2:**
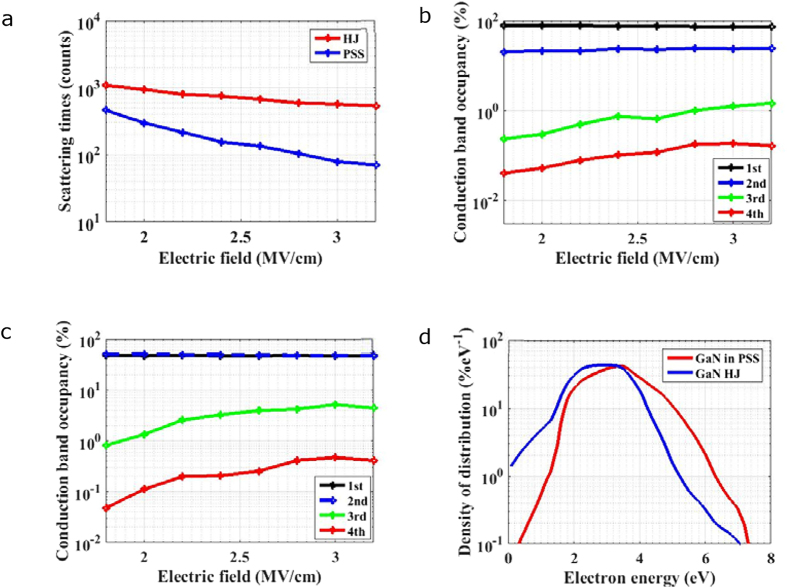
Electron transport properties for AlN (10 nm)/GaN (10 nm) PSS APD and GaN HJ APD. (**a**) *S*_***t***_ simulated under different electric field for PSS APD and HJ APD in 300 K. (**b**,**c**) Conduction band occupancy of electron in (**b**) GaN HJ APD and (**c**) GaN layers in GaN/AlN PSS APD under different electric field in 300 K. (**d**) Energy distribution curves of HJ APD and PSS APD under electric field of 1.8 MV/cm.

The averaged scattering times before electron reaches the ionization threshold energy (*S*_*t*_) of GaN HJ APD and GaN/AlN PSS APD are simulated and shown in Fig. 2a. It can be seen from the figure that the *S*_*t*_ of both PSS APD and HJ APD decrease with electric field. The descent degree of *S*_*t*_ for PSS under electric field ranging from 1.8 to 3 MV/cm is obviously higher than that for HJ APD. In addition, *S*_*t*_ for PSS is much less than that for HJ, especially in high electric field region.

Subsequently, energy states of electron during transport are monitored in FB-EMC process in GaN layers of GaN/AlN PSS APD and GaN HJ APD. Figure 2b,c show the band occupancy of each CB for (b) HJ APD and (c) GaN in PSS APD, respectively. In HJ APDs, electrons mainly distribute in first CB, while low portion occupies the higher order CBs. But in PSS APDs, the occupancy of the first CB is much reduced, while more electrons locate in higher order CBs. Furthermore, electron occupancy portions as a function of electron state energy in HJ APD and PSS APD are calculated and plotted in Fig. 2d (under electric field of 1.8 MV/cm). Thus, by aid of PSS, electrons in GaN could occupy more high energy portion.

### Temperature dependence of transport property

**Figure 3:**
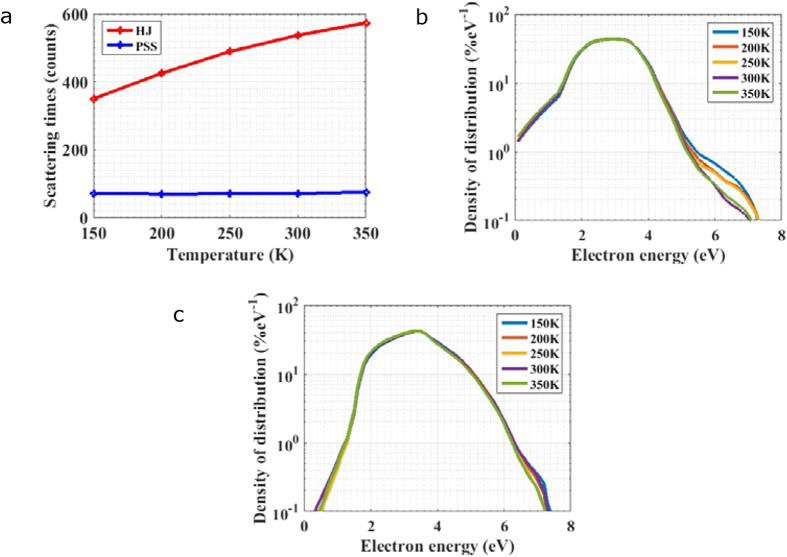
Temperature dependent properties for electron transport. (**a**) Temperature dependent properties of ***S***_***t***_ for HJ APD and PSS APD under electric field of 3.2 MV/cm. (**b,c**) Temperature dependent properties of energy distribution curves for (**b**) HJ APD and (**c**) PSS APD under electric field of 1.8 MV/cm.

Then, the temperature-dependent features are investigated. From Eqs 3, 4, 5, it can be seen that 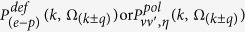 has a positive relationship with temperature. As *S*_*t*_ in PSS is much reduced compared with HJ APD, it can be predicted that the electron transport features in PSS APD will show weaker temperature dependence than in HJ APD. As shown in Fig. 3a, *S*_*t*_ simulated under an electric field of 3.2 MV/cm increases with temperature significantly in HJ APD, while *S*_*t*_ seems insensitive to temperature in PSS APD. Energy distributions are also simulated under different temperatures for HJ APD and PSS APD, as plotted in Fig. 3b,c, respectively. The occupancy of high energy potion in HJ APD varies drastically under different temperatures ranging from 150 to 350 K, while in PSS APD, energy distribution in the high energy states ranging from 4.5 to 7 eV possesses much weaker temperature dependence. Therefore, the temperature stability of energy occupancy in PSS APD is higher than that in HJ APD.

### Carriers’ ionization coefficients and temperature dependent features

**Figure 4:**
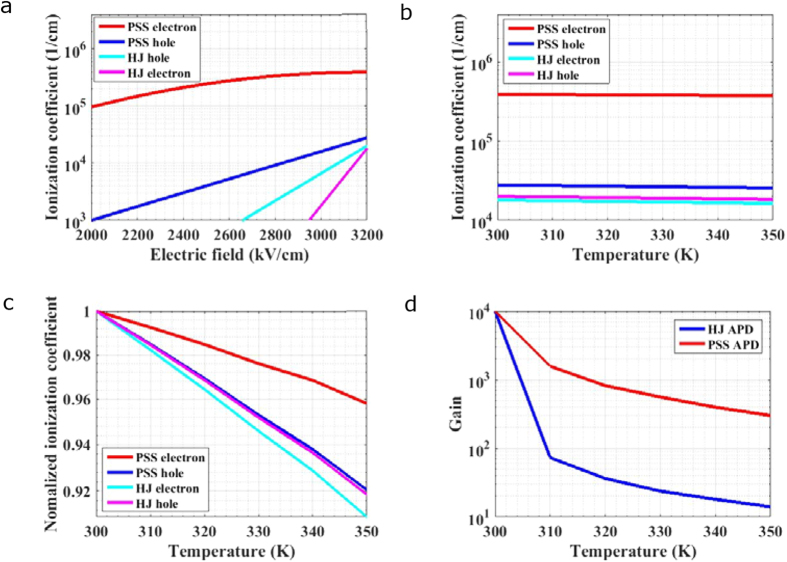
Carriers’ ionization coefficients and temperature dependent features for PSS APD and HJ APD. (**a**) Ionization coefficients of electron and hole under various electric field for PSS APD and HJ APD in 300 K. (**b**) Temperature dependence for carrier’s ionization coefficient for PSS APD and HJ APD under electric field of 3.2 MV/cm. (**c**) Temperature dependent ionization coefficients in (**b**) are normalized to the values under 300 K. (**d**) A comparison of the descent degree for gains with temperature between HJ APD and PSS APD.

Ionization coefficients and their temperature-dependent features in GaN (10 nm)/AlN (10 nm) PSS APD and HJ APD are compared. Firstly, ionization coefficients for electron and hole in PSS APD and HJ APD are calculated and plotted in Fig. 4a. Electron ionization coefficient of PSS APD is greatly enhanced compared with that of HJ APD. On the other hand, although there is a 0.7 eV valence band offset between AlN and GaN, no deep separated valleys exist in valence bands and hence hole still experiences high frequency of scatterings. Thus ionization coefficient for hole of PSS APD is not increased as much as that for electron. In other words, the difference between electron ionization coefficient and hole ionization coefficient is also significantly enlarged in PSS APD. It can be seen that electron ionization coefficient saturates at an extremely high level of 3.96 × 10^5^/cm under electric field over 3 MV/cm, significantly enhanced from the value about 10^3^/cm for electron in HJ APD. While hole ionization coefficient changes a bit around 10^4^/cm. Temperature dependence is investigated in electron and hole transport for PSS APD and conventional HJ APD. Ionization coefficients calculated at various temperatures under electric field of 3.2 MV/cm are plotted in Fig. 4b. In order to characterize their temperature dependence clearly, the values of ionization coefficients for electrons and holes of PSS APD and HJ APD are normalized to the value at 300 K and shown in Fig. 4c. As the scattering rate increases with temperature, the value of ionization coefficients declines with temperature. However, because the total scattering rate during electron transport in PSS APD is much reduced, the decline of ionization coefficient with temperature for electron in PSS APD is much slower than those for electron and hole in HJ APD or hole in PSS APD.

The temperature dependence of gain is also investigated. In order to compare the temperature dependence of multiplication performance reasonably, the electric fields for PSS APD and HJ APD are chosen to be 2.7 and 3.2 MV/cm, respectively, to fulfill that their gains are both 10^4^ under the same temperature of 300 K. Gains calculated under 300~350 K temperatures are plotted in Fig. 4d. The gains for both PSS APD and HJ APD decrease with the increase of temperature, but the temperature sensitivity of the latter is obviously stronger than that of the former. Especially when gains are high and ionization happens with high probability, the gain variation for PSS APD from 300 K to 310 K is about 20% lower than that for HJ APD. According to the analysis mentioned above, owing to the improvement of temperature stability for electron’s ionization coefficient, the temperature stability for multiplication gain rises. Therefore, PSS APD holds strong adaptation for wider application range, especially for applications under volatile temperature.

**Figure 5:**
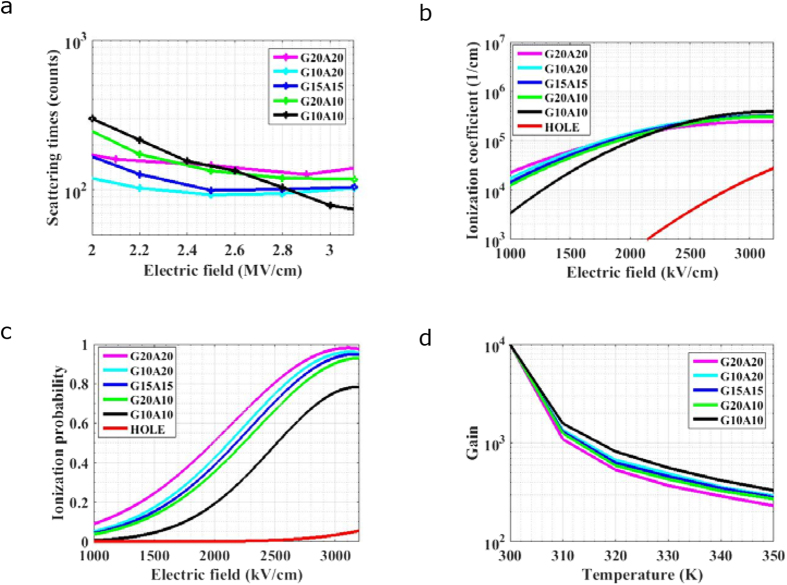
The influences of period length and layer thickness on ionization performance and temperature stability. (**a**) ***S***_***t***_ simulated under different electric fields at 300 K. (**b**) Ionization coefficients of electron and hole under various electric fields for PSS APDs at 300 K. (**c**) Ionization probability at each period for electron and hole under various electric fields at 300 K. (**d**) Temperature-dependent gains for different structures.

Finally, how period length and layer thickness impact on ionization performance and temperature stability is investigated. Three kinds of period lengths (20 nm, 30 nm, 40 nm) are taken into consideration. And a further refined study on three 30-nm period length samples is also carried out with the GaN thicknesses of 10 nm, 15 nm and 20 nm in each period, respectively. Thus, there are five samples of PSS APD with each period containing GaN (10 nm)/AlN (10 nm), GaN (10 nm)/AlN (20 nm), GaN (15 nm)/AlN (15 nm), GaN (20 nm)/AlN (10 nm) and GaN (20 nm)/ AlN (20 nm), labeled as G10A10, G10A20, G15A15, G20A10 and G20A20, respectively. Firstly, *S*_*t*_ is simulated under different electric field for PSS APDs with these five samples at 300 K as shown in Fig. 5a. *S*_*t*_ decreases with the increase of electric field for all samples. However, the descent rate for larger period length is lower and *S*_*t*_ increases with the increase of thickness for GaN within the same period length of 30 nm. Generally, the higher *S*_*t*_ is, the more sensitive to temperatures it is for APDs. Electrons’ ionization coefficients are simulated and shown in Fig. 5b. The ionization coefficients of holes for different samples are taken the same as G10A10 sample shown as red line as a simplification because PSS impacts little on hole ionizations. It can be seen that, increasing period length or increasing the proportion of AlN at each period in a specific range contributes to enhancing the ionization coefficient under low electric field. The probability of carriers triggering ionizations at each period for different samples are shown in Fig. 5c. The electric field required for PSS APD with thicker period length or larger proportion of AlN in each period to achieve the same ionization probability becomes lower. For example, to achieve a high ionization probability of 60%, the electric fields required for G20A20, G10A20, G15A15, G20A10, G10A10 are 2.15, 2.27, 2.33, 2.34 and 2.67 MV/cm, respectively. Lower electric field is preferred because the ionization coefficient ratio is higher. Finally, temperature-dependent gains for different structures are shown in Fig. 5d. The simulation is taken under the assumption that the periodicities of all samples are 20. Different electric fields are chosen to maintain the same gain of 10000 under 300 K, which are 2.3, 2.4, 2.5, 2.6 and 2.9 MV/cm for samples G20A20, G10A20, G15A15, G20A10 and G10A10, respectively. The corresponding values of *S*_*t*_ read from Fig. 5a are 159, 145, 100, 93 and 90, respectively. As the temperature stability deteriorates with the increase of *S*_*t*_, it can be seen in Fig. 5d that although making period length longer contributes to ionization coefficient ratio, the temperature stability suffers a bit reduction. Generally, electron in PSS APD with the thicker period length requires a lower electric field to gather enough energy to trigger ionizations. However, the transport time under such a low electric field will be longer and *S*_*t*_ increases consequently. On the other hand, increasing the AlN proportion at each period in a specific range facilitates in both ionization coefficient ratio and temperature stability. Considering the Γ valley depths of GaN and AlN are both 2 eV and their CB offset is also 2 eV, in order to gather the 5.3-eV ionization threshold energy^14^ of GaN with scattering degree as low as possible, the electron should always transport in Γ valleys of GaN and AlN until it achieves 4-eV energy and the additional 1.3-eV energy should be better gathered in AlN rather than in GaN. Under a fixed period of 30 nm, since the electric field in this discussion is around 2 MV/cm, if GaN is thicker than 10 nm, the energy gathered by electron in GaN will exceed 2 eV when it enters AlN. The extra energy beyond 2-eV is gathered at the cost of non-ignorable inter-valley scatterings in GaN, which could be better gathered in Γ valley of AlN with much less scatterings. Thus, samples G15A15 and G20A10 have the higher *S*_*t*_ than sample G10A20, and exhibit the lower temperature stability.

## Conclusion

In summary, decreasing scattering degree for electron transport is found to be necessary for realizing high temperature stability and high ionization efficient APD. By analyzing ionization coefficient, reducing DOS is found to be an essential way to achieve this goal. GaN/AlN PSS APD with deep Γ valley and large conduction band offset is systematically investigated. Compared with GaN HJ APD, *S*_*t*_ is significantly reduced, and more electrons distribute in high energy states, leading to a dramatically enhanced electron ionization coefficient. The influence of temperature on electron transport is also weakened, resulting in the less temperature dependent of electron ionization coefficient and multiplication gain in PSS APD. Moreover, thicker period length is found favorable to ionization coefficient ratio but a bit harmful for improving temperature stability, while increasing the proportion of AlN at each period in a specific range is favorable to both ionization coefficient ratio and temperature stability.

## Methods

### Full band ensemble Monte Carlo method

We give a quantitative analysis on the transport property and temperature stability based on full-band-ensemble-Monte-Carlo (FB-EMC) simulations, which is commonly accepted as a precise method to analyze the electron transport features in APD^5^,^15^,^16^,^17^,^18^. In this work, full band data in the whole Brillouin zone (BZ) of both GaN and AlN is calculated numerically by Empirical Pseudopotential Method^19^,^20^,^21^,^22^. Wave function is expanded by 183 ordered Bloch function. Four CBs are taken into consideration for both AlN and GaN. 1536000 and 276480 equally spaced sampling points are taken in the first BZ for the first CB and each of the other CBs, respectively. Then, scattering rates (consisting of the most important scattering types of deformation scattering and polar phonon scattering) between individual states in the whole BZ are solved with Fermi-Golden rules described by Eqs 3–4^5^,^23^. The data of ionization rates are referred from ref. 24, wherein the ionization rates corresponding with energies are given. As ionization event is a relative low probability event compared with other scattering mechanisms, we take a simplification that all the states with the same energy hold the same ionization rates. Finally, an ensemble Monte Carlo simulations are taken on electron transport in GaN/AlN PSS APD and GaN HJ APD, respectively. The period of GaN/AlN is 10/10 nm and cycle number is infinite. For simplification, interface scatterings and polarization field are not taken into account. During an electron getting through the GaN/AlN interfaces, energy conservation is fully observed, while momentum conservation is fulfilled as long as possible by selecting the final state closest to the initial one. For the electron with energy lower than 2-eV CB offset^13^ arriving at the GaN/AlN interface, it is reset to the bottom of 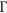 valley in AlN.

### Gain calculation

Multiplication gain initialed by an incident electron (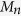) can be calculated by^25^


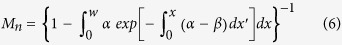


where 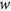 represents the multiplication region width, 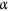 and 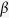 represent ionization coefficient for electron and hole, respectively. Dead space effect isn’t taken into consideration because 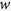 is taken as 400 nm, much longer than the dead space width (about 20 nm)^26^.

Calculating ionization probability from ionization coefficient

The probability (*P*) for carriers triggering ionization in one period can be calculated by


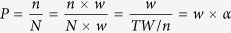


where 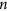 represents the average number of ionization events counted in a PSS sample with N periods. The total width of the sample is *TW* and the period length is *w*. 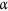 represents ionization coefficient.

## Additional Information

**How to cite this article**: Zheng, J. *et al.* Low-temperature-dependent property in an avalanche photodiode based on GaN/AlN periodically-stacked structure. *Sci. Rep.* **6**, 35978; doi: 10.1038/srep35978 (2016).

## Figures and Tables

**Figure 1:**
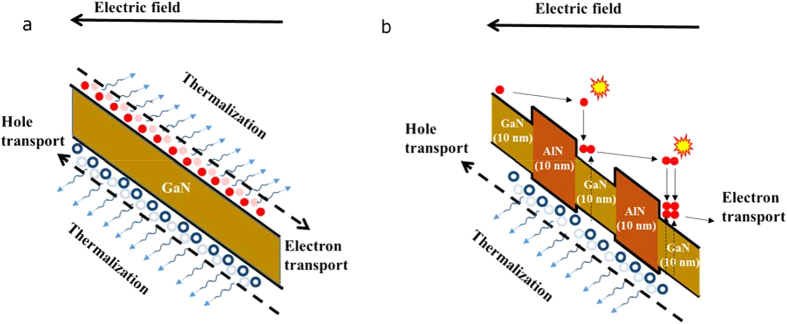
Motivation of the PSS structure. (**a**) In conventional HJ APD, most of the energy drawn from electric field is thermalized during electron and hole transport. (**b**) In PSS APD, the scattering degree during electron transport is greatly reduced.
